# DiffCircaPipeline: a framework for multifaceted characterization of differential rhythmicity

**DOI:** 10.1093/bioinformatics/btad039

**Published:** 2023-01-19

**Authors:** Xiangning Xue, Wei Zong, Zhiguang Huo, Kyle D Ketchesin, Madeline R Scott, Kaitlyn A Petersen, Ryan W Logan, Marianne L Seney, Colleen McClung, George Tseng

**Affiliations:** Department of Biostatistics, Graduate School of Public Health University of Pittsburgh, Pittsburgh, PA 15213, USA; Department of Biostatistics, Graduate School of Public Health University of Pittsburgh, Pittsburgh, PA 15213, USA; Department of Biostatistics, University of Florida, Gainesville, FL 32603, USA; Translational Neuroscience Program, Department of Psychiatry, University of Pittsburgh School of Medicine, Pittsburgh, PA 15219, USA; Translational Neuroscience Program, Department of Psychiatry, University of Pittsburgh School of Medicine, Pittsburgh, PA 15219, USA; Translational Neuroscience Program, Department of Psychiatry, University of Pittsburgh School of Medicine, Pittsburgh, PA 15219, USA; Department of Pharmacology and Experimental Therapeutics, Boston University School of Medicine, Boston, MA 02118, USA; Translational Neuroscience Program, Department of Psychiatry, University of Pittsburgh School of Medicine, Pittsburgh, PA 15219, USA; Translational Neuroscience Program, Department of Psychiatry, University of Pittsburgh School of Medicine, Pittsburgh, PA 15219, USA; Department of Biostatistics, Graduate School of Public Health University of Pittsburgh, Pittsburgh, PA 15213, USA

## Abstract

**Summary:**

Circadian oscillations of gene expression regulate daily physiological processes, and their disruption is linked to many diseases. Circadian rhythms can be disrupted in a variety of ways, including differential phase, amplitude and rhythm fitness. Although many differential circadian biomarker detection methods have been proposed, a workflow for systematic detection of multifaceted differential circadian characteristics with accurate false positive control is not currently available. We propose a comprehensive and interactive pipeline to capture the multifaceted characteristics of differentially rhythmic biomarkers. Analysis outputs are accompanied by informative visualization and interactive exploration. The workflow is demonstrated in multiple case studies and is extensible to general omics applications.

**Availability and implementation:**

R package, Shiny app and source code are available in GitHub (https://github.com/DiffCircaPipeline) and Zenodo (https://doi.org/10.5281/zenodo.7507989)

**Supplementary information:**

Supplementary data are available at *Bioinformatics* online.

## 1 Introduction

Circadian rhythms are 24-h oscillations of behavioral and biological processes that adapt life to the diurnal daylight cycle. The circadian rhythms regulate thousands of genes whose rhythmicity status and pattern are unique to tissue and cell types (Takahashi, 2017). The disruption of the process is associated with the development of various diseases (Logan and McClung, 2019), and thus there is increasing interest in circadian studies designed with contrasting conditions to reveal molecular mechanisms of rhythm-related physiological processes and disease etiology.

Although there are numerous rhythm detection algorithms, including parametric, flexible non-parametric or periodogram, all current differential rhythmicity (DR) methods are based on cosinor fitting (Cornelissen, 2014). The cosinor model $Y_{i}=A\;\text{cos}(\frac{2\pi }{P}(t_{i}-\phi ))+M+\epsilon_{i}$ (*P* = 24 h for circadian rhythm) assumes a sinusoidal relationship between the biomarker *Y* and Zeitgeber Time (ZT) *t*. It naturally defines multiple rhythmicity parameters and facilitates their differential analysis, including differential phase (Δϕ), amplitude (ΔA), MESOR (ΔM) and rhythm fitness ($\Delta R^{2}$, the coefficient of determination representing the proportion of variance explained by the circadian pattern). These four differential characteristics and their combinations are referred to as multifaceted DR characteristics.

Currently, DR methods either test for a combined shift in *A* and ϕ [e.g. HANOVA by Thaben and Westermark (2016), DODR by Thaben and Westermark (2016), LimoRhyde by Singer and Hughey (2019), compareRhythm by Parsons *et al.* (2020) and dryR by Weger *et al.* (2021)], or they test for *A*, ϕ, and *M* separately [e.g. Circacompare by Parsons *et al.* (2020) and diffCircadian by Ding *et al.* (2021)]. However, the former cannot identify changes in individual parameters, whereas the latter risks inflating Type I error rates when testing multiple parameters. Additionally, DODR and diffCircadian test for changes in rhythm fitness by comparing the noise level ($\Delta \sigma^{2}$), which as we show in Supplementary Material S4.2.2, is biologically less meaningful than testing $\Delta R^{2}$.

Moreover, the DR tests are only meaningful when the biomarkers are rhythmic in at least one group. The procedures of categorizing biomarkers to types of rhythmicity (TOR) by the groups they are rhythmic include AIC/BIC-based model selection (used by compareRhythm and dryR) and Venn diagram analysis (used by Circacompare and diffCircadian), whereas the latter lacks consideration for multiple testing and is heavily criticized statistically (Hughes *et al.*, 2017; Pelikan *et al.*, 2022).

Given developed methods and limitations in existing papers and software packages, there is an urgent need for an integrated pipeline for detecting DR biomarkers with systematic multifaceted characterization. Our proposed ‘DiffCircaPipeline’ will be the first comprehensive framework to meet this gap. Beyond a pure bioinformatics workflow, DiffCircaPipeline contains multiple methodological innovations for completing the integrative framework. Firstly, we developed a selective sequential model selection (SSMS) procedure that has an accurate Type I error control for categorizing TOR for biomarkers. Secondly, we demonstrated that testing DR fitness with $\Delta R^{2}$ is biologically more informative than testing $\Delta \sigma^{2}$, and we proposed a new likelihood ratio test, which is more efficient than the permutation test used in Chen *et al.* (2016). Finally, when multiple parameters are of interest, we proposed a global DR parameter test (similar to LimoRhyde) and followed by a new *post hoc* individual parameter test. DiffCircaPipeline is available in both R package and user-friendly R shiny app with visualization tools to help the users perform the analyses interactively.

## 2 Results

**Fig. 1.**
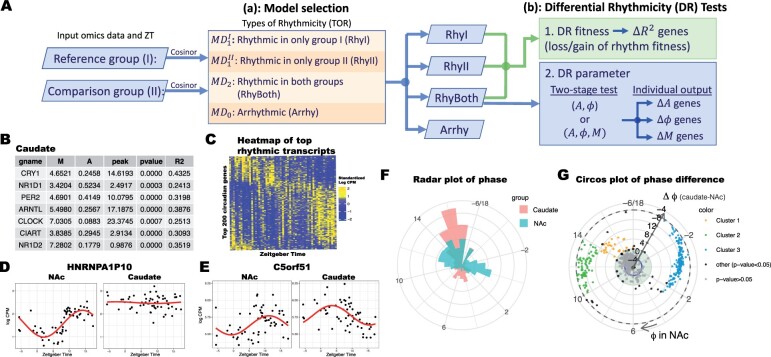
Workflow and example outputs in the nucleus accumbens (NAc, the reference) versus caudate (comparison) of Case Study. 1. (**A**) Flowchart of DiffCircaPipeline. Step (a): Model selection to categorize biomarkers to four types of rhythmicity. Step (b): Two DR tests: DR fitness ($\Delta R^{2}$) and DR parameters (Δϕ, ΔA, ΔM). (**B**) Table of the rhythmic canonical clock genes in caudate. (**C**) Heatmap of the top 200 circadian genes detected in caudate (CPM stands for counts per million). (**D, E**) Scatter plots with fitted rhythmic curve in a DR fitness ($\Delta R^{2}$) gene HNRNPA1P10 ($R^{2}=0.6$ in NAc and $R^{2}=0.01$ in caudate) and in a differential phase (Δϕ) gene C5orf51 (ϕ=11.3 in NAc and ϕ=3.5 in caudate). (**F**) Radar plot for distributions of phase for NAc and caudate in RhyBoth rhythmic genes. (**G**) Circos plot for phase difference between NAc and caudate. Each dot represents a gene. The angular axis is phase ϕ in NAc and the radius is the phase difference Δϕ between caudate and NAc

Figure 1A presents an overview of the DiffCircaPipeline workflow. The pipeline is designed for DR analysis of two contrasting groups, denoted as Groups I (reference) and II (comparison). Before input, omics data are preprocessed by conventional practices (e.g. for RNA-Seq data, preprocessing includes filtering out non-expressed transcripts, normalization and log transformation). When detecting DR in the three sinusoidal curve parameters (Δϕ, ΔA, ΔM), only biomarkers that are rhythmic in both groups are qualified. In contrast, for biomarkers with DR fitness ($\Delta R^{2}$), biomarkers rhythmic in one or more groups would qualify. As a result, Step (a) in Figure 1A first performs cosinor model fitting in each group separately. For example, Figure 1B shows the parameter estimates of the circadian core clock genes in the brain region caudate in Case Study 1 to be introduced later. A SSMS procedure (G’Sell *et al.*, 2016) is applied to categorize biomarkers into four TORs: (i) rhythmic only in Group I (RhyI); (ii) rhythmic only in Group II (RhyII); (iii) rhythmic in both groups (RhyBoth); and (iv) arrhythmic in both groups (Arrhy). This procedure ensures that false positive rates are accurately controlled, and different DR tests are performed in each TOR category. We evaluated two kinds of false positive rates: (i) identifying an Arrhy biomarker as the other three categories and (ii) identifying a RhyI/RhyII biomarker as RhyBoth. We showed that our SSMS procedure has the most accurate Type I error control in both scenarios compared to several conventional procedures including the Venn diagram, Akaike information criterion/Bayesian information criterion (AIC/BIC)-based model selection and adaptively weighted Fisher. Supplementary Section S2.2 describes all these methods in detail, and Supplementary Section S3.1 provides the simulation-based evaluation.

After determining TOR, DR analyses are performed in Step (b) of Figure 1A for DR fitness ($\Delta R^{2}$) and DR curve parameters (Δϕ, ΔA, ΔM). For DR fitness test, biomarkers rhythmic in one or more groups (RhyI, RhyII and RhyBoth) will be tested. In Supplementary Section S2.3, we show that $\Delta R^{2}$ is approximately equivalent to the difference in signal-to-noise ratio (ΔSNR=Δ(A/σ)) and propose a likelihood ratio test based on ΔSNR. We also validate that overall $\Delta R^{2}$ identifies biologically more meaningful biomarkers than using $\Delta \sigma^{2}$ in Supplementary Section S4.1.3.Figure 1D shows scatter plots and fitted rhythmic curves of HNRNPA1P10 as a ‘DR fitness’ gene with loss of rhythmicity, in which the gene is a circadian biomarker in NAc ($R^{2}=0.6$) but is not rhythmic in caudate ($R^{2}=0.01$) We note that genes detected by $\Delta R^{2}$ are different from RhyI and RhyII from the model selection. Supplementary Section S4.1.3 provides two counterexamples for illustration.

Next, only biomarkers rhythmic in both groups are eligible for DR parameter test including Δϕ, ΔA, ΔM. Existing methods (CircaCompare and diffCircadian) that perform all these tests do not consider controlling for multiple testing. In Step (b) of Figure 1A, DiffCircaPipeline solves this problem by performing a two-stage test. The first-stage global test detects the overall change of multiple parameters [e.g. (ϕ, *A*) or (ϕ, *A*, *M*)], and the second-stage *post hoc* test tests each parameter individually. Specifying parameters of interest before the test is important to avoid inflation of the Type I error due to multiplicity. Details of existing methods, the proposed two-stage test, and their simulation evaluations are provided in Supplementary Sections S2.4 and S3.3. Figure 1E shows scatter plots and fitted rhythmic curves of C5orf51 as a differential phase gene when testing for change in (ϕ, *A*) simultaneously. This gene is a circadian biomarker for both brain regions but has a peak time of ZT 11.3 in NAc and ZT 3.5 in caudate.

## 3 Four case studies and conclusions

In the first case study, DiffCircaPipeline is applied for DR analysis in transcriptomic data between nucleus accumbens (NAc) and caudate in the human postmortem brain of subjects without psychiatric or neurological disorders (Ketchesin *et al.*, 2021). Among 14 754 genes after preprocessing, SSMS procedure identifies 1487 RhyI genes, 1638 RhyII genes and 546 RhyBoth genes. We performed differential phase tests to the 546 RhyBoth genes and found 274 genes with significant changes in phase with *P*-value < 0.05 and 254 genes with false discovery rate (FDR) < 5%.

DiffCircaPipeline produces multiple informative visualization plots to assist rhythmicity comparison. Figure 1C shows a heatmap of the top 200 rhythmic transcripts identified in caudate ordered by phase. Scatter plots of gene expression across ZT with fitted cosinor curves are used to demonstrate gene-specific rhythm patterns. Figure 1D shows HNRNPA1P10 with differential rhythm fitness while Figure 1E shows C5orf51 with differential phase. To summarize differential phase results, we use radar plots (Fig. 1F) to show the distributions of phase for RhyBoth genes, which reveals evident peak time difference in NAc and caudate. In addition, circos plots in Figure 1G displays the phase of reference (NAc) on the angular axis and phase difference (Δϕ) on the radius. The circos plot shows three clusters of differential phase genes. The green cluster is enriched in cell division-related processes, such as DNA replication, histone binding and G2/M checkpoint. The blue cluster is enriched in pathways related to ribosome components, apoptosis and membrane-associated processes. These findings imply different cellular activities occur in NAc and caudate at different times, which may be associated with the functional difference between the two brain regions. Details are available in Supplementary Section S4.1.

In Case Study 2, we demonstrated our pipeline by comparing brain transcriptomic data between subjects with schizophrenia and unaffected comparison (UC) subjects. With a *P*-value cutoff of 0.05, we identified 268 RhyI genes, 252 RhyII genes and 12 RhyBoth genes. Circadian rhythm signaling is the most enriched pathway of rhythmic genes in UC subjects (*P* = 0.0085), with canonical clock genes as the top predicted upstream regulators. Next, we performed DR fitness tests for the 532 genes and identified 97 genes with a significant change of *R*^2^ (*P*-value < 0.1). ‘Calcium signaling’ (*P* = 0.0031) and ‘LPS/IL-1 Mediated Inhibition of RXR Function’ (*P* = 0.0027) are the most enriched pathway for genes with increased *R*^2^ and decreased *R*^2^ correspondingly. Both pathways are found to be associated with schizophrenia. Damped or amplified oscillations are evident on the scatter plots of genes from the two pathways (Supplementary Section S4.2).

Finally, we applied our pipeline to DNA methylation data to show its generalizability to other omics data (Case Study 3), and to a five-gene real-time RT-PCR data to demonstrate a small data application (Case Study 4). Details are available in Supplementary Sections S4.3 and S4.4.

The current software has at least two limitations. Firstly, the pipeline assumes that the circadian oscillation follows a sinusoidal curve, which is currently the only existing method to simultaneously characterize MESOR, phase and amplitude but a more flexible model may be developed in the future. Secondly, our pipeline is only designed for two-group comparison and cannot easily extend to a multi-group scenario. Methods to overcome these limitations are directions of future development.

*Financial Support:* This work was supported by the National Institutes of Health [K01MH128763 to KK, R01DA051390 to MS and RL, R01MH111601 to CM and GT, P50DA046346 to CM] and the University of Pittsburgh Center for Research Computing, RRID:SCR_022735, through the resources provided. Specifically, this work used the HTC cluster, which is supported by NIH award number S10OD028483.

*Conflict of Interest*: All authors declare no conflict of interest.

## Supplementary Material

btad039_Supplementary_Data
