# Supplementary material for: DiffCircaPipeline: a framework for multifaceted characterization of differential rhythmicity
Source: Bioinformatics. 2023 Jan 19;39(1):btad039. doi: 10.1093/bioinformatics/btad039 (PMC9889843; doi:10.1093/bioinformatics/btad039)
Supplement: btad039_Supplementary_Data [file btad039_supplementary_data.pdf]

Supplementary Material "DiffCircaPipeline: A framework for multifaceted characterization of differential rhythmicity"

December 15, 2022

1 Overview

This supplementary file provides details of statistical methods and validations that are not included in the main text. For R/shiny software tutorial, please refer to the software manuals at Github. This section introduces the model notations and the recommended methods and alternatives we provide in the pipeline. A more detailed description of methods and the justification of the recommendations are shown in Section 2 corroborated by simulation results in Section 3. Finally, we demonstrate the pipeline with four case studies (Section 4).

1.1 The study design and notations

DiffCircaPipeline is designed for studies that compare two contrasting groups for differential rhythmicity, denoted as group I (reference) and II (comparison). The analyses require the input of biological measurements from samples and corresponding sample collection times. The time of day is converted to Zeitgeber time (ZT) based on the onset of light (Figure 1), where ZT0 corresponds to sunrise. On average, ZT0 is often referred to 6 AM but the actual sunrise time depends on the date and location (latitude and longitude), for which we have provided a function *DCP_getZT* for convenient conversion.

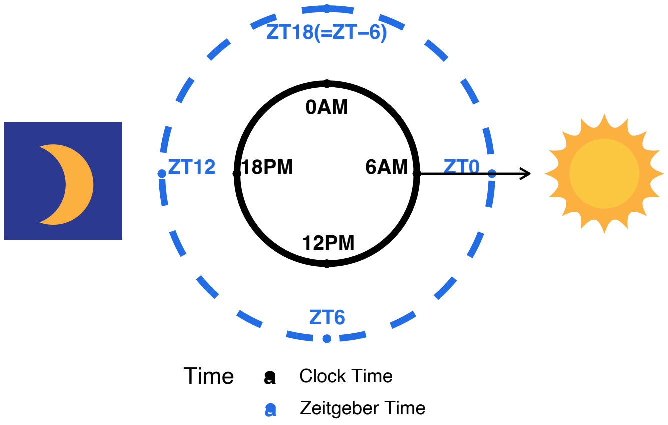

Figure 1: A comparison between clock time and Zeitgeber time. In this diagram, we assume that sunrise time (the start of the light cycle) is 6 AM and sunset time (the start of the dark cycle) is 18 PM. In reality, these time points change with location and day in a year.

We denote the input biological data as $Y_1 \in \mathcal{R}^{G\times N_1}$ and $Y_2 \in \mathcal{R}^{G\times N_2}$, with the Zeitgeber time of the samples $t_1 \in \mathcal{R}^{N_1}$ and $t_2 \in \mathcal{R}^{N_2}$. Here N_1 and N_2 are sample sizes of group I and group II, respectively, and G is the number of biological features (e.g., genes or methylation features). The cosinor model:

$$Y_i = A\cos(\omega(t_i - \phi)) + M + \epsilon_i,\ \epsilon_i \sim N(0, \sigma^2)$$

characterizes the gene rhythmicity with four parameters: amplitude (A), phase (ϕ), Midline Estimating Statistic Of Rhythm (MESOR, M) and the noise level (σ). The peak time equals phase under this formula, so the two terms will be used interchangeably. The constant $\omega = \frac{2\pi}{P}$ is pre-specified, and $P = 24$ hours is used for circadian rhythmicity although different P could also be used (e.g., 12-hour rhythmicity). Specifically, the signal to noise ratio SNR $= A/\sigma$ describes the strength of rhythm, which is equivalent to the goodness of fit statistic R^2 under mild conditions (see Proposition 2 in Section 2.3). The subscript of parameters indicates the group number, i.e. A_1 is the amplitude of group I and A_2 is for group II. Parameters without the subscript are shared by both groups, i.e. $A = A_1 = A_2$.

1.2 Recommended methods and alternatives

Before input to DiffCircaPipeline, data should be processed appropriately. Examples include filtering out low-expressed features, removing low-quality samples or outliers, normalization between samples, log transformation, etc. The processing procedure varies between data platforms and thus is not included in DiffCircaPipeline.

1.2.1 Categorization of types of rhythmicity

In step (a) (Figure 1A(a) in the main text), we categorize biomarkers into four types of rhythmicity (TOR): arrhythmic in both groups (Arrhy), rhythmic in only group I (RhyI), rhythmic in only group II (RhyII), and rhythmic in both groups (RhyBoth). This step is essential because the differential rhythm fitness test is only meaningful if a biomarker is rhythmic in at least one group, while the differential rhythm parameter test is only meaningful with biomarkers rhythmic in both groups.

Denoting the rhythmicity p-values from the two groups as p_1 and p_2, DiffCircaPipeline provides three methods for this purpose (more available methods and detailed descriptions can be found in Section 2.2):

- (Default) SSMS with Sidak.FS (Selective sequential model selection with Sidak adjustment and ForwardStop). This method sequentially selects the rhythmic groups for each biomarker by applying a pre-specified cutoff to p_1 and p_2 (details in Section 2.2).
- VDA (Venn diagram analysis). This method compares p_1 and p_2 with a given cutoff separately to get lists of biomarkers that are rhythmic in each group. Then the overlap of the two biomarker lists are considered RhyBoth.
- AW (Adaptively weighted Fisher's method). This method calculates a combined p-value from p_1 and p_2 indicating the existence of rhythm in at least one of the groups. At the same time, weights (0 or 1) are estimated for the two groups to indicate TOR (i.e., the weight is (1, 1) for RhyBoth biomarkers and (1, 0) for RhyI biomarkers).

We recommend the SSMS with Sidak.FS method based on its better control of two types of type I error. The first one is TypeI_0, the misclassification of Arrhy biomarker versus any other types of biomarkers, and the second is cTypeI_1, the misclassification of RhyI or RhyII versus RhyBoth. Among the listed methods, Sidak.FS is the only one that controls both types of type I error well (see Section 3.1 for simulation results).

1.2.2 Differential rhythm fitness test

The differential rhythm fitness test is performed for biomarkers rhythmic in at least one group. In DiffCircaPipeline, we characterize the rhythm fitness with the goodness of fit statistic R^2, which is approximately equivalent to SNR (A/σ) (see Section 2.3 Proposition 2). Three tests are available for testing differential R^2:

- (Default) likelihood ratio test,
- permutation test,
- bootstrap test.

We describe these tests in detail in Section 2.3 and benchmark them in Section 3.2. The likelihood ratio test is recommended due to the most consistent type I error control over varying SNRs and under the existence of differential parameters. The computation speed of the likelihood ratio test is also the fastest compared to the two sampling-based methods.

In the literature, differential rhythmicity fitness has been tested by whether $R_1^2 = R_2^2$ (Ketchesin et al. (2021)) or by $\sigma_1^2 = \sigma_2^2$ (Ding et al. (2021)). We find that a difference in R^2 is more meaningful and more concordant with a difference in oscillation amplitude and rhythmicity p-value. However, a difference in σ^2 does not imply changes in other rhythmicity characteristics. As a result, only testing for R^2 is pursued in DiffCircaPipeline. Justification of the choice to test the differential R^2 rather than the differential σ^2 can be found in Section 4.1.3.

1.2.3 Differential rhythm parameter test

The differential rhythm parameter test is performed for only RhyBoth biomarkers to detect the difference in the three rhythm curve parameters ($A_1 = A_2$, $\phi_1 = \phi_2$ or $M_1 = M_2$). If one is only interested in testing one of the parameters, the likelihood ratio test in diffCircadian package (Ding et al. (2021)) will be performed. However, if the change in more than one parameter is of interest, we perform the two-stage differential rhythmicity test with first-stage global test followed by second-stage post hoc individual parameter tests. Two global tests are available: (1) change in A and ϕ together, or (2) change in A, ϕ, and M.

Performing a two-stage differential rhythmicity test rather than multiple individual tests helps avoid the type I error inflation from multiplicity. Section 2.4 describes the tests and Section 3.3 validates the type I error control by simulations.

2 Method

2.1 Estimation and testing of cosinor model

We recall the cosinor model Cornelissen (2014):

$$Y_i = A\cos(\omega(t_i - \phi)) + M + \epsilon_i, \tag{1}$$

where $\epsilon_i \sim N(0, \sigma^2)$ and $\omega = 2\pi/P$ is pre-defined. For circadian rhythmicity, we have $P = 24$ hours.

Applying the trigonometric conversion $\cos(x+y) = \cos x \cos y - \sin x \sin y$, Equation 1 transforms to

$$Y_i = \beta_1 \cos(\omega t_i) + \beta_2 \sin(\omega t_i) + M,$$

with $\beta_1 = A\cos\omega\phi$ and $\beta_2 = A\sin\omega\phi$. With $\cos(\omega t_i)$ and $\sin(\omega t_i)$ being constants transformed from t, β_1 and β_2 can be readily estimated by ordinary least square regression. Then we calculate the rhythm parameters by:

$$\hat{A} = \sqrt{\hat{\beta_1}^2 + \hat{\beta_2}^2}, \text{ and } \hat{\phi} = \frac{1}{\omega}(\arctan(-\hat{\beta}_2/\hat{\beta}_1) + K\pi),$$

where K is an integer.

To detect a rhythmic biomarker, we test the significance of rhythmicity with hypotheses $H_0 : A = 0$ and $H_0 : A \neq 0$ with the F statistics

$$F = \frac{(\text{TSS} - \text{RSS})/2}{\text{RSS}/(N-3)} \sim F(2, N-3), \tag{2}$$

where RSS is the residual sum of squares and TSS is the total sum of squares: $\text{RSS} = \sum_{i=1}^n \left(Y(t_i) - \hat{Y}(t_i)\right)^2$ and $\text{TSS} = \sum_{i=1}^n \left(Y(t_i) - \bar{Y}(t_i)\right)^2$. Here $\hat{Y}(t_i) = \hat{A}\cos(\omega(t_i - \hat{\phi})) + \hat{M}$, $\bar{Y}(t_i) = \sum_i^N Y(t_i)/N$ (N is the sample size). With the critical level α, the null hypothesis is rejected when $F > F_{1-\alpha,2,N-3}$ ($F_{q,a,b}$ is the qth percentile of F distribution with degrees of freedom a and b).

2.2 Categorization of types of rhythmicity

After fitting the expression data with the cosinor model for the two groups separately, we categorize the biomarkers into four types of rhythmicity (TOR): arrhythmic in both groups (Arrhy), rhythmic in only group I (RhyI), rhythmic in only group II (RhyII), and rhythmic in both groups (RhyBoth).

For this purpose, one commonly used method is Venn diagram analysis (VDA), which is inappropriate due to the lack of concern for multiplicity and arbitrary cutoff choice. It is also pointed out in Pelikan et al. (2021) that VDA tends to classify Arrhy and RhyBoth biomarkers to RhyI or RhyII. The authors then proposed two schemes in the package compareRhythm: hypothesis testing and model selection (described below) to classify the biomarkers into five categories (the corresponding category in DiffCircaPipeline are listed in the parenthesis and will be used thereafter): arrhythmic (Arrhy), gain of rhythmicity (RhyII), loss of rhythmicity (RhyI), same rhythmicity (RhyBoth), change of rhythmicity (RhyBoth). Note that "same rhythmicity" or "change of rhythmicity" from compareRhythm are concatenated to "RhyBoth" in our paper due to difference in definition of "change of rhythmicity".

In DiffCircaPipeline we propose to apply and generalize the selective sequential model selection (SSMS) G'Sell et al. (2016) procedure. To illustrate, we build a nested model path starting from the simplest model where the biomarker is not rhythmic in either groups (MD_0). The next model in the path has the biomarker rhythmic in only one group (MD_1), and the full model has the biomarker rhythmic in both groups (MD_2).Then we use stopping rules to select a parsimonious model on the model path. Figure 2 summarizes the construction of the model path according to the order of rhythmicity p-values p_1 and p_2. The models in Figure 2 are:

$$
\begin{aligned}
MD_0 &: Y_i = g_{1i}(M_1 + \epsilon_{1i}) + g_{2i}(M_2 + \epsilon_{2i}), \\
MD_1^I &: Y_i = g_{1i}(M_1 + A_1 \cos(\omega(t_i - \phi_1)) + \epsilon_{1i}) + g_{2i}(M_2 + \epsilon_{2i}), \\
MD_1^{II} &: Y_i = g_{1i}(M_1 + \epsilon_{1i}) + g_{2i}(M_2 + A_2 \cos(\omega(t_i - \phi_2)) + \epsilon_{2i}), \text{ and} \\
MD_2 &: Y(t_i) = g_{1i}(M_1 + A_1 \cos(\omega(t_i - \phi_1)) + \epsilon_{1i}) + g_{2i}(M_1 + A_2 \cos(\omega(t_i - \phi_2)) + \epsilon_{2i}).
\end{aligned}
$$

Here $g_{1i} = 1$ and g_{2i} are index functions for if sample i is from group I or group II, correspondingly.

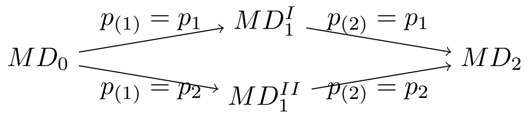

Figure 2: Two possible model paths. Here p_1 and p_2 denote rhythmicity p-values from group I and group II, respectively. $p_{(1)}$ and $p_{(2)}$ is the smaller and the larger one of p_1 and p_2.

Rhythmicity p-values p_1 and p_2 are generated by testing MD_0 against MD_1^I in group I data and testing MD_0 against MD_1^{II} in group II data separately. We use $p_{(1)} = \min(p_1, p_2)$ and $p_{(2)} = \max(p_1, p_2)$ to denote the smaller and the larger one of them respectively, and denote $p_{(1)}$ and $p_{(2)}$ as single step p-values for step one and step two. Next, stopping rules are applied to select a parsimonious model. Denote k as the step that we stop at, e.g., when the true category is RhyBoth, the step two model MD_2 is true and $k = 2$. Given a critical value α, we consider the following two stopping rules:

- BasicStop (BS) (Marcus et al. (1976)) selects the largest model with single-step p-value smaller than α:
$$\hat{k}_B = \max\{k : p_k < \alpha\}$$
- ForwardStop (FS) (default): considers all the preceding single-step p-values:
$$\hat{k}_F = \max\{k : -\frac{1}{k}\sum_{i=1}^{k} \log(1 - p_i) \leq \alpha\}$$

Note that using rhythmicity p-values as the single step p-values is anti-conservative because we keep selecting the smallest p-value, which is biased without a pre-determined path(Taylor and Tibshirani (2015)). So the critical value of the stopping rules is adjusted for the sequential steps using Sidak (Šidák (1967)): $\alpha^* = 1 - (1-\alpha)^{1/d}$, where $d = 2$ is the number of single-step p-values.

In Section 3.1, we will perform simulation and compare the performance of the selective sequential model selection method with ForwardStop (Sidak.FS) or BasicStop (Sidak.BS) with VDA, the two compareRhythm methods (hypothesis testing and model selection), and the AW-Fisher method used by Ketchesin et al. (2021):

- Selective sequential model selection methods (Sidak.BS and Sidak.FS): The model path and single-step p-values are computed as described above. Then we tested BS and FS for model selection with a Sidak adjusted critical value.
- Venn Diagram Analysis (VDA): Compare p_1 and p_2 to α separately and use Venn Diagram to decide assignment of the four TOR categories.
- Hypothesis testing (RAIN.DODR): Identify the rhythmic biomarkers with RAIN (Thaben and Westermark (2014)) for both groups, then perform DODR (Thaben and Westermark (2016)) for biomarkers rhythmic in more than one group. The biomarkers are categorized with the following rules:
  - Not rhythimic in neither I nor II by RAIN: Arrhy
  - Rhythmic in only I by RAIN and changes rhythmicity by DODR: RhyI
  - Rhythmic in only II by RAIN and changes rhythmicity by DODR: RhyII
  - Rhythmic in either group but not changes rhythmicity by DODR: RhyBoth
  - Rhythmic in both groups by RAIN: RhyBoth
- Model selection (ModSel.AIC and ModSel.BIC): Select the best model that correspond to the four categories based on AIC or BIC criteria.
- AW-Fisher (AW): Apply the p-value combination method AW-Fisher (Huo et al. (2020)) to p_1 and p_2 to calculate a combined p-value. biomarkers with a combined p-value larger than α are Arrhy. For biomarkers with a combined p-value smaller than α, assign the biomarkers to RhyI, RhyII, or RhyBoth by the AW-Fisher weights $(1,0)$, $(0,1)$ and $(1,1)$.

2.3 Differential rhythm fitness test

For rhythmic biomarkers in at least one group, we ask if there is a difference in the data's rhythm fitness to the cosinor model across groups. The rhythm fitness is characterized by the goodness-of-fit statistics R^2:

$$R^2 = 1 - \frac{\text{RSS}}{\text{TSS}}.$$

In Chen et al. (2016) the difference in R^2 is tested using permutation test. In the next subsections, we propose a likelihood ratio test and a bootstrap test for the same purpose. All the methods are compared in section 3.2. The hypothesis for testing R^2 is:

$$H_{0,R^2} : R_1^2 = R_2^2 = R_c^2;\ H_{A,R^2} : R_1^2 \neq R_2^2.$$

2.3.1 Likelihood ratio test (LR)

It is difficult to derive the exact analytical form for differential R^2 test. However, with Proposition 1- 2, we proved that difference in R^2 can be approximated by difference in the signal to noise ratio (SNR $= A/\sigma$). We then perform likelihood ratio test for equal SNR under the null hypothesis:

$$H_{0,\text{SNR}} : A_1/\sigma_1 = A_2/\sigma_2;\ H_{A,\text{SNR}} : A_1/\sigma_1 \neq A_2/\sigma_2.$$

Proposition 1.

$$\mathbb{E}\left(R^2\right) = 1 - \mathbb{E}\left(\frac{RSS}{TSS}\right) \rightarrow 1 - \frac{\mathbb{E}(RSS)}{\mathbb{E}(TSS)},\ as\ n \rightarrow +\infty$$

Proof. Note that $\frac{1}{n}RSS \xrightarrow{p} \mathbb{E}(\frac{RSS}{n})$ and $\frac{1}{n}TSS \xrightarrow{p} \mathbb{E}(\frac{TSS}{n})$, thus by continuous mapping theorem,

$$\frac{RSS}{TSS} \xrightarrow{p} \frac{\mathbb{E}(RSS)}{\mathbb{E}(TSS)}.$$

Taking expectation for both sides with bounded R^2 ($R^2 \leq 1$), we get

$$\mathbb{E}\left(\frac{RSS}{TSS}\right) \rightarrow \frac{\mathbb{E}(RSS)}{\mathbb{E}(TSS)}.$$

□

Proposition 2. *Under the condition that the sampled time points for the two groups are the same, testing R^2 is equivalent to testing $\frac{A}{\sigma}$.*

Proof. Note that $\mathbb{E}(RSS) = \sigma^2(n-r)$, where $r = 3$ is the number of parameters in equation 1. Denote $\hat{y_i} = A\cos(w(t_i - \phi)) + C$.

$$\begin{aligned}
\mathbb{E}(TSS) &= \mathbb{E}\left(\sum_{i=1}^{n}(y_i - \bar{y})^2\right) \\
&= \mathbb{E}\left(\sum_{i=1}^{n} y_i^2\right) - n\mathbb{E}(\bar{y}^2) \\
&= \sum_{i=1}^{n}\left(\mathbb{V}(y_i) + (\mathbb{E}y_i)^2\right) - n\left(\mathbb{V}(\bar{y}) + (\mathbb{E}\bar{y})^2\right) \\
&= n\sigma^2 + \sum_i (\hat{y_i})^2 - \sigma^2 - n\left(\frac{1}{n}\sum_i \hat{y_i}\right)^2 \\
&= (n-1)\sigma^2 + \frac{1}{n}\sum_{i\neq j}(\hat{y_i} - \hat{y_j})^2 \\
&= (n-1)\sigma^2 + \frac{A^2}{n}\sum_{i\neq j}\left(\cos(w(t_i - \phi)) - \cos(w(t_j - \phi))\right)^2.
\end{aligned}$$

$$\begin{aligned}
\mathbb{E}\left(R^2\right) &= 1 - \mathbb{E}\left(\frac{RSS}{TSS}\right) \\
&\rightarrow 1 - \frac{\mathbb{E}(RSS)}{\mathbb{E}(TSS)} \\
&= 1 - \frac{\sigma^2(n-r)}{(n-1)\sigma^2 + \frac{A^2}{n}\sum_{i\neq j}\left(\cos(w(t_i - \phi)) - \cos(w(t_j - \phi))\right)^2} \\
&= 1 - \frac{n-r}{n-1+n\frac{A^2}{\sigma^2}\frac{1}{n^2}\sum_{i\neq j}\left(\cos(w(t_i - \phi)) - \cos(w(t_j - \phi))\right)^2}.
\end{aligned}$$

If $\sum_{i\neq j}\left(\cos(w(t_i - \phi)) - \cos(w(t_j - \phi))\right)^2$ is the same for both groups, i.e. the sampled time points are the same, then $\mathbb{E}\left(R_1^2\right) \approx \mathbb{E}\left(R_2^2\right)$ if $\frac{A_1}{\sigma_1} = \frac{A_2}{\sigma_2}$.

□

2.3.2 Permutation test

The permutation test is performed by randomly shuffling the samples along with zeitgeber time between two groups for B times. The bth shuffling produces the pseudo data: $Y_1^{(b)}$, $t_1^{(b)}$, $Y_2^{(b)}$ and $t_2^{(b)}$. Then we fit the pseudo data with cosinor model and calculate R_g^2 for biomarker g ($R^2 = 1 - \frac{\text{RSS}}{\text{TSS}}$). The empirical null distribution for ΔR^2 is then $(\Delta R_g^2)^{(b)} = (R_{2g}^2)^{(b)} - (R_{1g}^2)^{(b)}, b = 1, 2, ..., B$. The p-value for an observed $\Delta \hat{R}_g^2$ is calculated as $\frac{\sum_1^B |\Delta R_g^2| > |(\Delta R_g^2)^{(b)}|}{B}$. Note that B should be a relatively large number and we use $B = 1000$ throughout the paper.

2.3.3 Bootstrap test

Another non-parametric test we propose is the bootstrap test. Similarly, we sample with replacement within the group for B times to produce the pseudo data and calculate the empirical null distribution of ΔR_g^2. The p-value for an observed $\Delta \hat{R}_g^2$ is $\frac{\sum_1^B |\Delta R_g^2| > |(\Delta R_g^2)^{(b)}|}{B}$.

2.4 Two-stage differential rhythm parameter test

For RhyBoth biomarkers, one might be interested in testing if any of the rhythmicity parameters (A, ϕ) or the MESOR are statistically different between the two groups. The DiffCircaPipeline performs a two-stage DR parameter test, which comprises a global test followed by post hoc individual parameter tests. We provide two commonly-used integrated hypotheses for the first-stage global tests:

$$\text{Differential } A \text{ or } \phi : H_{0,(A,\phi)} : A_1 = A_2 \text{ and } \phi_1 = \phi_2; H_{A,(A,\phi)} : \text{complement of } H_{0,(A,\phi)} \quad (3)$$

$$\text{Differential } A, \phi \text{ or } M : H_{0,(A,\phi,M)} : A_1 = A_2, \phi_1 = \phi_2 \text{ and } M_1 = M_2; H_{A,(A,\phi,M)} : \text{complement of } H_{0,(A,\phi,M)} \quad (4)$$

To test differential A or ϕ (Equation 3), F test between the following null and alternative models are performed:

$$\begin{aligned} &H_{0,(A,\phi)} : Y(t_i) = M_1 + M_d \times g_{2i} + A\cos(\omega(t_i - \phi)) + \epsilon; \\ &H_{A,(A,\phi)} : Y(t_i) = M_1 + M_d \times g_{2i} + g_{1i} \times A_1 \cos(\omega(t_i - \phi_1)) + g_{2i} \times A_2 \cos(\omega(t_i - \phi_2)) + \epsilon_i, \end{aligned}$$

where $g_{2i} = 1$ if sample i belongs to group II and $g_{2i} = 0$ if otherwise. In the null model only difference in MESOR between the two groups is allowed while in the alternative model the extra cosinor term accounts for the difference in both A and ϕ.

Similarly, the null and alternative models to test A, ϕ and M (Equation 4) are

$$\begin{aligned} &H_{0,(A,\phi,M)} : Y(t_i) = M + A\cos(\omega(t_i - \phi)) + \epsilon_i; \\ &H_{A,(A,\phi,M)} : Y(t_i) = g_{2i} \times (M_1 + A_1 \cos(\omega(t_i - \phi_1))) + g_{2i} \times (M_1 + A_2 \cos(\omega(t_i - \phi_2))) + \epsilon_i, \end{aligned}$$

If the global F tests reject the null, we then perform the second-stage post hoc tests for individual parameters with a Sidak-adjusted p-value threshold for the number of parameters tested. If the post hoc tests reject the individual null, conclusions can be made on individual differential parameters.

When only one parameter is of interest (e.g., differential phase), the corresponding individual test using the diffCircadian package will suffice. Note, in Ding et al. (2021), it is shown that the permutation test, Circacompare, and the LR tests are equally powerful with well controlled type I error. So the choice of using diffCircadian here is by convenience without further validations. In section 3.3, we will benchmark the performance of the two-stage tests with type I error control and power analysis with simulation data.

3 Numerical evaluation and justification of method choices in the pipeline

Simulations in this section provide justifications for recommended methods of each analytical step: (1) Sidak.FS for categorizing TOR; (2) likelihood ratio test for detecting DR fitness biomarkers (ΔR^2); (3) the two-step differential rhythm parameter test for detecting multiple differential paramters.

3.1 Evaluating methods for classifying types of rhythmicity categories

For evaluations in this subsection, we will borrow the benchmarks of type I error from selective sequential model selection (Fithian et al. (2015)). The aim is to select the simplest model that best describes the data's sampling distribution. Thus, a type I error occurs when we reject the lower model and accept an upper

model while the lower model is adequate. In the context of our classification question, such type I error occurs when: (1) an Arrhy biomarker ($k = 0$) is classified to be any other types ($\hat{k} = 1$ or $\hat{k} = 2$), denoted as

$$\text{TypeI}_0 = P(\hat{k} > 0 | k = 0);$$

(2) a RhyI or a RhyII biomarker ($k = 1$) is classified to RhyBoth ($\hat{k} = 2$), denoted as

$$\text{TypeI}_1 = P(\hat{k} = 2 | k = 1).$$

Note that, a RhyI biomarker could also be incorrectly classified to RhyII or Arrhy, where a wrong model rather than a correct model with redundant variables is selected. Thus it is more meaningful to discuss a conditional type I error given a selection of the correct model:

$$\text{cTypeI}_1 = P(\hat{k} = 2 | k = 1, \hat{MD}_1 = MD_1) \tag{5}$$

We then define the power as the probability of classifying biomarkers to their true category:

$$\text{Power}_1 = P(\hat{k} = 1, \hat{MD}_1 = MD_1 | k = 1, MD_1); \tag{6}$$

$$\text{Power}_2 = P(\hat{k} = 2 | k = 2). \tag{7}$$

3.1.1 Simulation setting

We simulate biomarker expression data from the cosinor model:

$$Y_i = M + A\cos(\omega(t_i - \phi)) + \epsilon_i, \epsilon_i \sim N(0, \sigma^2).$$

Rhythmic biomarkers are generated with SNR $= A/\sigma > 0$, while arrhythmic biomarkers are generated with $A = 0$, $\sigma = 1$. The method RAIN requires equal spaced time points. To compare all the methods with the same simulated data, we sampled data at time points 0, 4, 8, 12, 16, 20 (unit is hour), each repeated 5 times, which makes 30 samples in total for each group. Since M is independent from the rhythmicity signal, we draw M from $\text{UNIF}(5, 10)$ for both groups.

We simulated 10,000 Arrhy, RhyI, and RhyBoth biomarkers (RhyII is equivalent to RhyI, thus omitted) and compared the performance of different methods based on TypeI_0, cTypeI_1, Power_1 and Power_2. All the settings are then repeated 10 times for variance estimation. For RhyI biomarkers we further studied the impact of SNR: $A_1/\sigma_1 = 0.5, 0.6, 0.7, 0.8, 0.9, 1$ with fixed $\sigma_1 = 1$ and $\phi_1 = 0$. This range covers the SNR we observe from real human data. When SNR > 1, all the methods show high power (greater than 0.8) with well-controlled TypeI_0 and cTypeI_1 thus is not shown. For RhyBoth biomarkers we simulated the following variations to study the impact of different SNR, shifted phase and unbalanced SNR between groups:

- Same SNR: Both Groups have the same SNR with $A_1/\sigma_1 = A_2/\sigma_2 = 0.5, 0.6, 0.7, 0.8, 0.9, 1$ with fixed $\sigma_1 = \sigma_2 = 1$ and $\phi_1 = \phi_2 = 0$.
- Same SNR with different phase: Both Groups have the same SNR=1 but shifted phase with $A = \sigma = 1$; change $\phi_2 = 4, 6, 8, 10$, while fix $\phi_1 = 0$.
- Different SNR with same phase: Group I has fixed $\text{SNR}_1 = 1$ with $A_1 = \sigma_1 = 1$. For group II, we fix $A_2 = 1$ but change $\sigma_2 = 2.50, 1.67, 1.25, 0.83, 0.67$ ($\text{SNR}_2 = 0.40, 0.60, 0.80, 1.20, 1.50$, correspondingly). The two groups have the same phase.

3.1.2 TypeI_0 and cTypeI_1

With a nominal level $\alpha = 0.05$, AW-Fisher, both Sidak.FS and Sidak.BS control the TypeI_0 perfectly with minimal variance. -based model selection roughly controls TypeI_0, while VDA, RAIN.DODR, and AIC-based model selection all show a significant inflation of TypeI_0 (Figure 3A).

Next we evaluated cTypeI_1 restricted to methods that show good control of TypeI_0 (Figure 3B). AW-Fisher and ModSel.BIC both fail to control cTypeI_1. Sidak.FS shows well controlled cTypeI_1. Sidak.BS is too conservative, but we still compare its power with Sidak.FS in the next section.

The poor performance of VDA in controlling TypeI_0 is expected due to its lack of concern for multiplicity, and its empirical type I error is consistent with the theoretical deviation $(1 - (1 - 0.95)^2 = 0.0975)$. RAIN.DODR is even more anti-conservative, which may be due to the excessive sensitivity of RAIN to identify rhythmic biomarkers with a non-parametric procedure. The AIC-based model selection is the most anti-conservative, which agrees with the long-observed fact that AIC tends to favor more complex models given the small penalty it gives to the number of parameters selected (Shen and Ye (2002), Aho et al. (2014)). In our case, this property translates into selecting more groups to be rhythmic. AW-Fisher is theoretically guaranteed to detect overall signal (i.e., if the biomarker is rhythmic in any groups), but has a poor finite sample performance in the weight estimation (i.e., which group is rhythmic), which is consistent with its perfect performance in controlling TypeI_0 but not cTypeI_1. Similarly, BIC's advantage of selecting the correct model is also not guaranteed with a finite sample (Aho et al. (2014)).

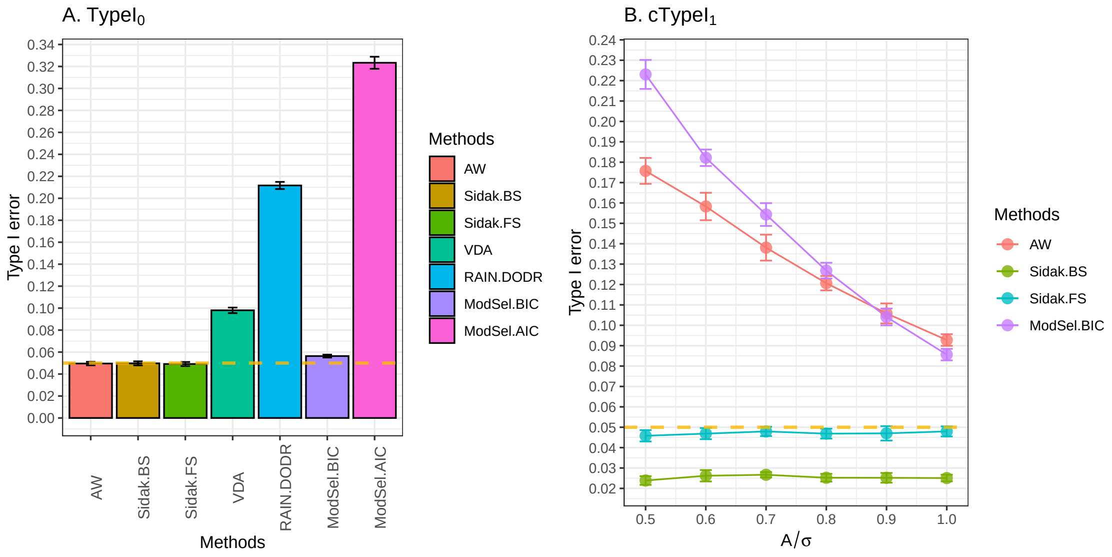

Figure 3: Type I error control of different methods of classifying the biomarker TOR. A. $\text{TypeI}_0 = P(\overline{\text{Arrhy}}|\text{Arrhy})$, B. $\text{cTypeI}_1 = P(\text{RhyBoth}|\text{RhyI and group I is selected})$.

3.1.3 Power analysis

When the biomarkers are rhythmic in only one group, Sidak.BS and Sidak.FS perform similarly with Power_1 increasing with SNR (Figure 4A). When biomarkers are rhythmic in both groups with the same SNR or different SNR, Sidak.FS is always higher in Power_2 than Sidak.BS (Figure 4B). Difference in phase does not impact Power_2 (Figure 4C).

3.1.4 Genome-wide false discovery rate of Sidak.FS

To control the genome-wide Type I error, the p-values from fitting MD_1^I and MD_1^{II} (Equation 2.2 and 2.2) for all the biomarkers are adjusted separately using the Benjamini-Hochberg Procedure (Benjamini and Hochberg (1995)) and then input to the selective sequential model selection procedure introduced in section 2.2.

We then extend the definition of false discovery rate (FDR) and expected discovery rate (EDR) to mirror those of type I error and power defined at the start of section 3.1:

- $\text{FDR}_0 = E[P(k = 0|\hat{k} > 0) \times P(\hat{k} > 0)]$. FDR of classifying Arrhy biomarkers to be rhythmic in any groups.
- $\text{FDR}_1 = E[P(k = 1|\hat{k} = 2) \times P(\hat{k} = 2)]$. FDR of classifying RhyI or RhyII biomarkers to RhyBoth.
- $\text{EDR}_1 = E[P(\hat{k} = 1|k = 1)]$. EDR of RhyI or RhyII biomarkers.

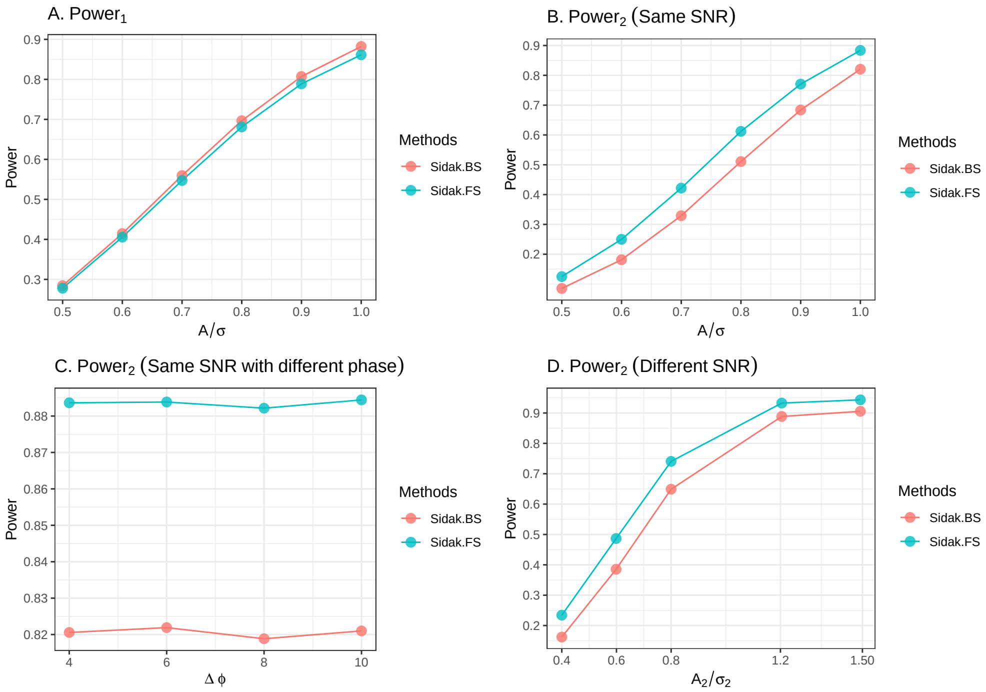

Figure 4: Power Analysis. A. $\text{Power}_1 = p(\hat{k} = 1|k = 1)$. B. $\text{Power}_2 = p(\hat{k} = 2|k = 2)$. Power_2 when both groups have same SNR and phase. C. Power_2 when the two groups have the same SNR but different phase. D. Power_2 when the two groups have the same phase ($\phi = 0$) but different SNR.

- $\text{EDR}_2 = E[P(\hat{k} = 2|k = 2)]$. EDR of RhyBoth biomarkers.

We simulated 10,000 biomarkers for each data, and the 10,000 biomarkers consists of following types of biomarkers and variations: (1) 9,000 Arrhy biomarkers with $\sigma = 1$; (2) 200 RhyI biomarkers with $A_1 = \sigma_1 = 1$; (3) 200 RhyII biomarkers with $A_2 = \sigma_2 = 1$; (4) 200 RhyBoth biomarkers with same parameters between groups ($A = \sigma = 1$ and $\phi = 0$); (5) 200 Rhyboth biomarkers with same SNR ($A = \sigma = 1$) but different phase ($\phi_1 = 0$ and $\phi_2 = 4$); (5) 200 RhyBoth biomarkers with different SNR ($A_1 = \sigma_1 = A_2 = 1$, $\sigma_2 = 1.25$) but same phase ($\phi = 0$). We repeated simulating this setting for 1,000 times for accurate benchmark. Note that we could have tried to distribute the types of biomarkers to mimic that in the real data we observe, but the real data may vary in other studies. So instead, we simulated most biomarkers to be arrhythmic, which is consistent with most real data, and evenly distribute the other types of biomarkers.

The proposed method controls FDR very well at the nominal level 0.05 for both FDR_0 and FDR_1 (Figure 5A) and achieves high genome-wide power (EDR) for all types of rhythmic biomarkers (Figure 5B).

3.2 Evaluating methods for differential rhythm fitness tests

In section 2.3 we proved that testing R^2 between two groups is approximately identical to testing $\text{SNR} = A/\sigma$ under mild conditions. As a result, we simulate the two groups with the same SNR to evaluate the type I error control of the compared methods and then different SNR for power analysis.

biomarkers with varying SNR are simulated to evaluate the impact of SNR level to type I error control. We fix $A = 3$ and varied σ to generate SNR at: $0.4, 0.6, 0.8, 1, 1.2, 1.5, 2, 2.5, 3, 3.5, 4$. This grid covers the empirical SNR from many human and mouse circadian data. To study the impacts of existence of differential parameters to the performance of differential rhythm fitness tests, we further simulated the following setting: (1) no differential parameters, where $M_1 = M_2 = 5, \phi_1 = \phi_2 = 0$; (2) differential M, where we fix $\phi_1 = \phi_2 = 0, M_1 = 5$ and vary $M_2 = 5.5, 6.25$; (3) differential ϕ, where we fix $M_1 = M_2 = 5, \phi_1 = 0$ and vary $\phi_2 = 12, 18$.

For power analysis we fix $\text{SNR}_1 = 3$ while changing $\text{SNR}_2 = 0.4, 0.6, 0.8, 1.2, 1.5, 2, 2.5, 3, 3.5, 4$, where we

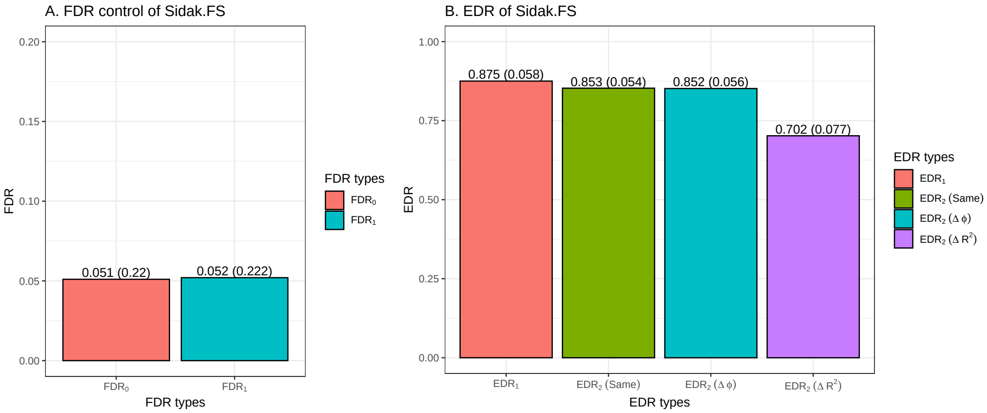

Figure 5: Genome-wide FDR and EDR: the number on the bars are mean (SD) from the simulation.

fix $A_1 = A_2 = 3$ for both groups and only change σ_2. Same variations with M and ϕ as above are simulated. All the settings for type I error control and power analysis are simulated with 10,000 biomarkers and 20 samples with time variables drawn from $UNIF(0, 24)$, and the procedure was repeated 10 times.

3.2.1 Type I error control

When the two groups have the same A, ϕ, and M as well as SNR, the type I error control varies with the shared SNR value for all three methods (Figure 6A). The type I error rate of the bootstrap test fluctuates wildly and is higher than the nominal level in most cases. The type I error rate increases slowly with SNR but is generally well controlled with the permutation test. With LR, the type I error is conservative with low SNR but is steady around the nominal level when SNR > 1. Also, the method LR has stable standard deviation (SD) for type I error rate, while both Bootstrap and Permutation methods have increasing SD with SNR.

Furthermore, when differential parameters exist, the type I error control of the permutation test varies drastically, while it is unaffected for LR and the bootstrap test (Figure 7). In summary, only the LR test controls type I error well under the nominal level with varying SNR values or presence of differential parameters and thus, it is adopted as the default method in DiffCircaPipeline.

3.2.2 Power analysis

Use $\text{SNR}_1 = 3$ as the baseline (the blue dotted line in Figure 6B), statistical power of the three tests increase when the effect size (ΔR^2) increases, while LR constantly shows the largest power.

3.3 Evaluating the two-stage differential parameter test

To benchmark, Type I error of the first-stage global test is defined as the probability of rejecting global null (Equation 3 or 4) given the null is true. Type I error of the second-stage post hoc test is the probability of falsely rejecting the corresponding individual parameter null after rejecting the global null.

3.3.1 Simulation setting

Only RhyBoth biomarkers are simulated to evaluate the DR parameter tests. Time points are generated from UNIF$(0, 24)$. We simulated the same data sets for testing $H_{0,(A,\phi)}$ and $H_{0,(A,\phi,M)}$ with only the testing procedures changed. In each simulation, 10,000 biomarkers under null or under alternative are simulated and are repeated 10 times for estimation of variance. In all the simulations we fix the noise level $\sigma = 1$.

For type I error evaluation, the parameters tested are set to be the same between the two groups. The following settings are simulated for the test of $H_{0,(A,\phi,M)}$: (1) fix $A = 3, \phi = 0$ and vary $M = 5, 6, 7$; (2) fix $A = 3, M = 5$ and vary $\phi = 0, 6, 12$; (3) fix $M = 5, \phi = 0$ and vary $A = 2, 3, 4$. For the test of $H_{0,(A,\phi)}$, same

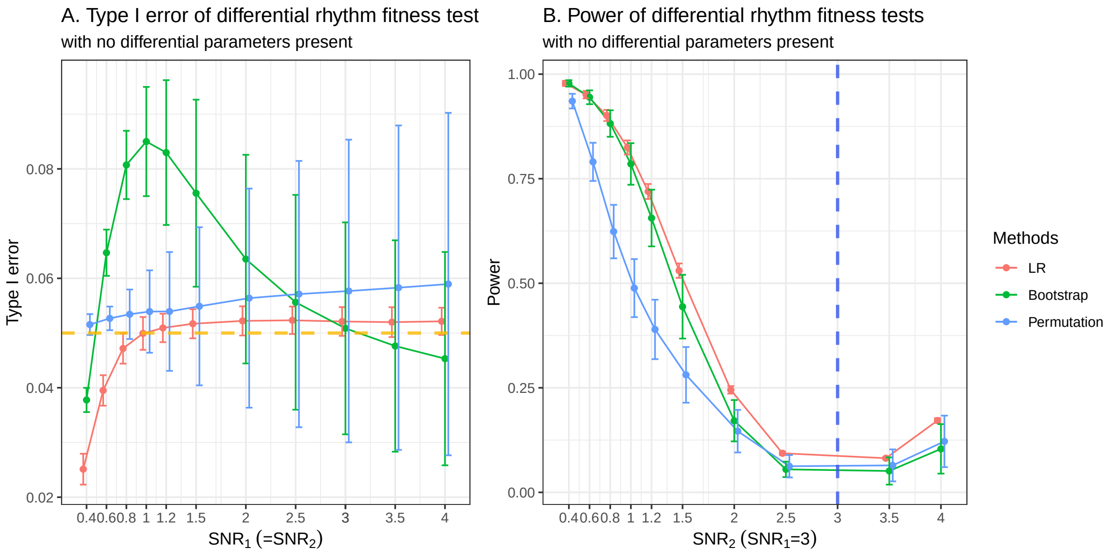

Figure 6: Type I error and Power analysis of differential rhythm fitness tests when there is no differential parameters present. A. Type I error control. B. Power.

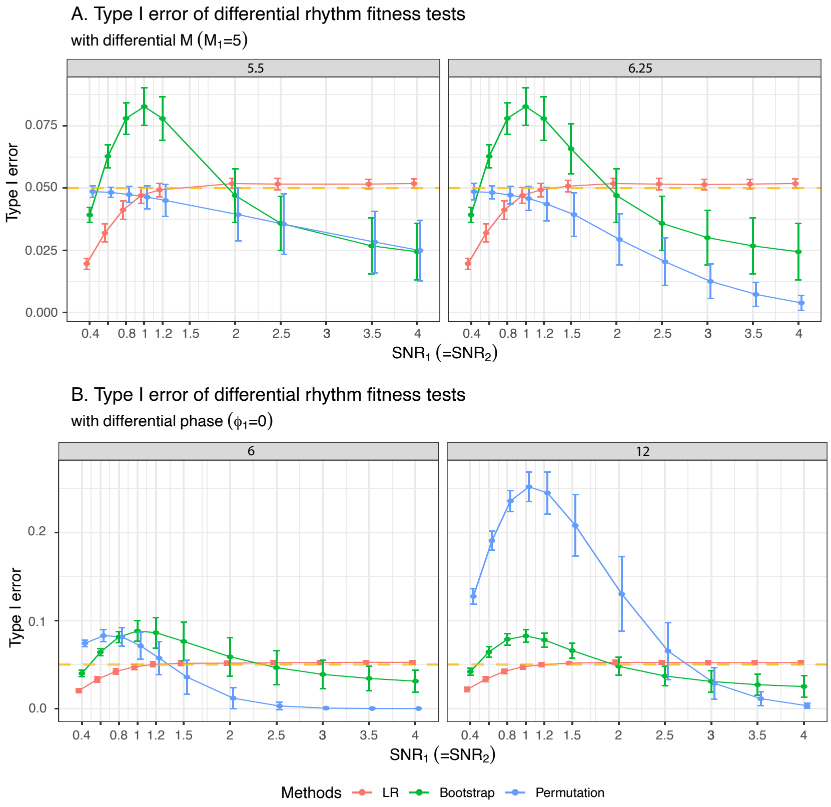

Figure 7: Type I error of differential rhythm fitness tests with A. differential M ($M_1 = 5$, $M_2 = 5.5$ or 6.25), B. differential phase ($\phi_1 = 0$, $\phi_2 = 6$ or 12).

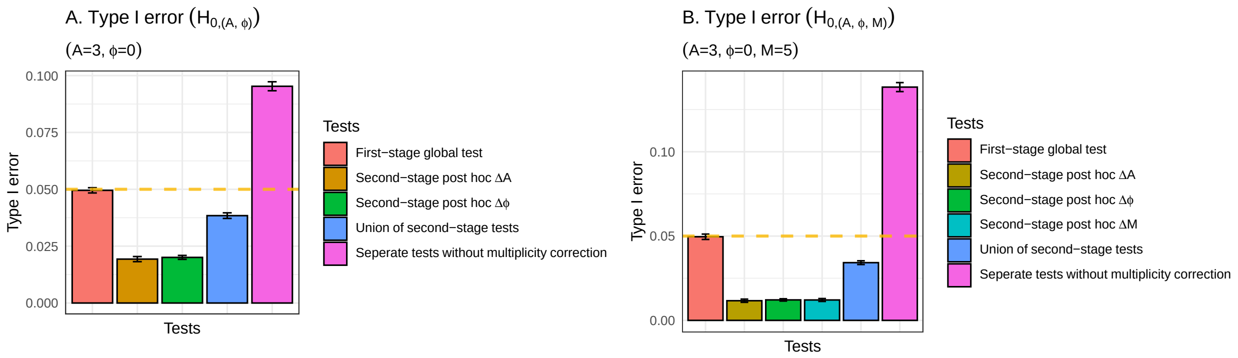

Figure 8: Type I error control of the two-stage differential rhythm parameter test.

variations in (2) and (3) are simulated while we further simulated cases where M is different between the two groups: fix $A = 3, \phi = 0, M_1 = 5$ and vary $M_2 = 6, 7$.

For power analysis, the parameters for group I are fixed: $A_1 = 3, \phi_1 = 0$ and $M_1 = 5$. The parameters for group II is changed one at a time: (1) fix $A_2 = 3, \phi_2 = 0$ and vary $M_2 = 5.5, 6, 6.5, 7$; (2) fix $A_2 = 3, M_2 = 5$ and vary $\phi_2 = 2, 4, 6, 8$; (3) fix $M_2 = 5, \phi_2 = 0$ and vary $A_2 = 3.5, 4, 4.5, 5$.

3.3.2 Type I error control

When the data are simulated under the null, the value of parameters does not affect the type I error, so only results at $A = 3, \phi = 0$ and $M = 5$ are shown. The first-stage global test controls the type I error rate perfectly at the nominal level in both testing scenarios (orange bars in Figure 8A and B). In the second-stage post hoc test, the union of the two or three post hoc tests is only slightly conservative (blue bars in Figure 8A and B). In contrast, if the parameter tests are performed separately without multiplicity correction, the test is very anti-conservative with inflated type I error to 10-15% (blue bars in Figure 8A and B).

4 Case studies

In this section, we illustrate the workflow of DiffCircaPipeline with three case studies to demonstrate the functionality and wide-applicability of the software. The first study compares the transcriptomic rhythmicity between two brain regions, nucleus accumbens (NAc) and caudate, focusing on healthy human subjects. In the second example, we study the differential rhythmicity between subjects with schizophrenia and unaffected comparison subjects in dorsal lateral prefrontal cortex (dlPFC) region in brain. Finally, we show that the DiffCircadianPipeline is applicable to general omics data by applying to a DNA methylation data set.

4.1 Case study one: rhythm comparison between brain regions with RNA-Seq data

Ketchesin et al. (2021) pairwisely compared the transcriptomic rhythmicity between three regions (NAc, caudate, and putamen). Here, we apply DiffCircaPipeline for DR between NAc and caudate only. The RNA-Seq data is quantified from the same cohort of 59 psychiatric-disorder-free subjects. The subjects' recorded time of death (TOD) was normalized to the zeitgeber time (ZT) scale and used as the expression time for biomarkers. The data is publicly available at Gene Expression Omnibus (GEO) with accession number GSE160521 and more details about the data could be found in Ketchesin et al. (2021).

4.1.1 Identification of types of rhythmicity

We used Sidak.FS to identify the TOR for the two brain regions and identified 546 RhyBoth genes, 1,487 RhyI genes and 1,638 RhyII genes at p-value cutoff 0.05 (Figure 9A). At FDR=30% cutoff, we identify 396 RhyBoth genes, 1,174 RhyI genes and 1,015 RhyII genes. With pathway enrichment analysis tool Metascape

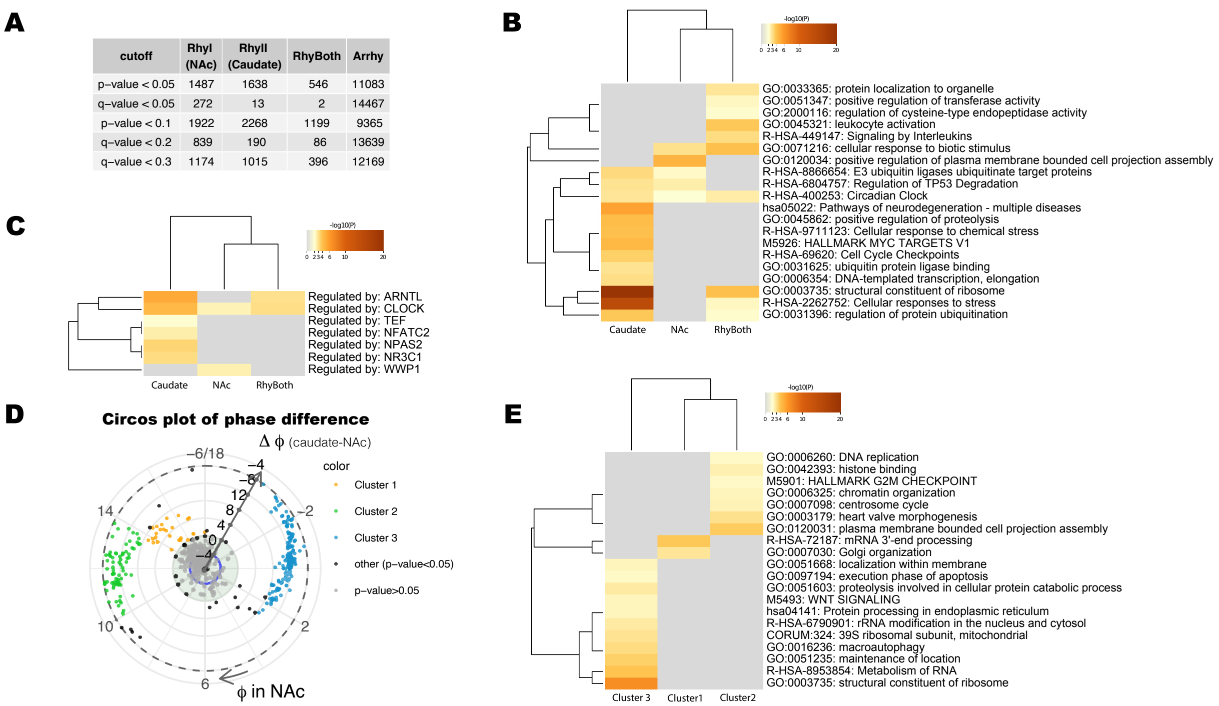

Figure 9: Circadian rhythmicity comparison result between nucleus accumbens (NAc) and caudate. A. Number of genes categorized to each TOR by different cutoffs. B. Top pathways enriched for each brain region. C. Upstream regulator enriched. D. Circos plots showing phase difference between the two brain regions. Each dot on the plot represents one RhyBoth gene. The angular axis is the gene's phase in NAc, and the radius is the phase difference comparing caudate to NAc. The radius axis is arranged such that the more interesting genes with peak differences greater than 4 hours are displayed at the outer circle for better visualization. The light-green-shaded area bounds the genes with a peak difference smaller than 4 hours. The non-grey genes have p-values smaller than 0.05 from the differential phase test (without multiple comparison correction for exploratory purpose). The differential phase genes of NAc and caudate are separated into three clusters by color: blue, yellow and green. E. Top pathways enriched for three clusters of differential phase genes.

(Zhou et al. (2019)), we performed pathway enrichment tests for all genes rhythmic in NAc (RhyI and RhyBoth), all genes rhythmic in caudate (RhyII and RhyBoth), and the RhyBoth genes. Within the 546 RhyBoth genes, we find enrichment in circadian clock and cell cycle regulation pathways (Figure 9B). The top predicted upstream regulators include the core clock genes like ARNTL, CLOCK, and NPAS2 (Figure 9C).

4.1.2 Genes with phase shift between two brain regions

We are interested in the genes that reach the peak expression at a different time in different brain regions. In Figure 9D, the peak time of RhyBoth genes between NAc and caudate are shown and the differential phase genes form three clusters, e.g., the green cluster peaks between ZT 10 to ZT 14 in NAc, and its peak time in caudate is around 8 hours earlier. Similar phase shifts also exist in the yellow cluster and the blue cluster. Pathway enrichment results in Figure 9E reveal that cluster 2 is involved in cell division-related processes like DNA replication, histone binding, G2/M checkpoint, chromatin organization, and centrosome cycle. Cluster 3 is enriched in pathways related to ribosome components, apoptosis, and membrane-associated processes. These findings imply that these cellular activities occur in NAc and caudate at different times, which may be associated with a functional difference between the two brain regions.

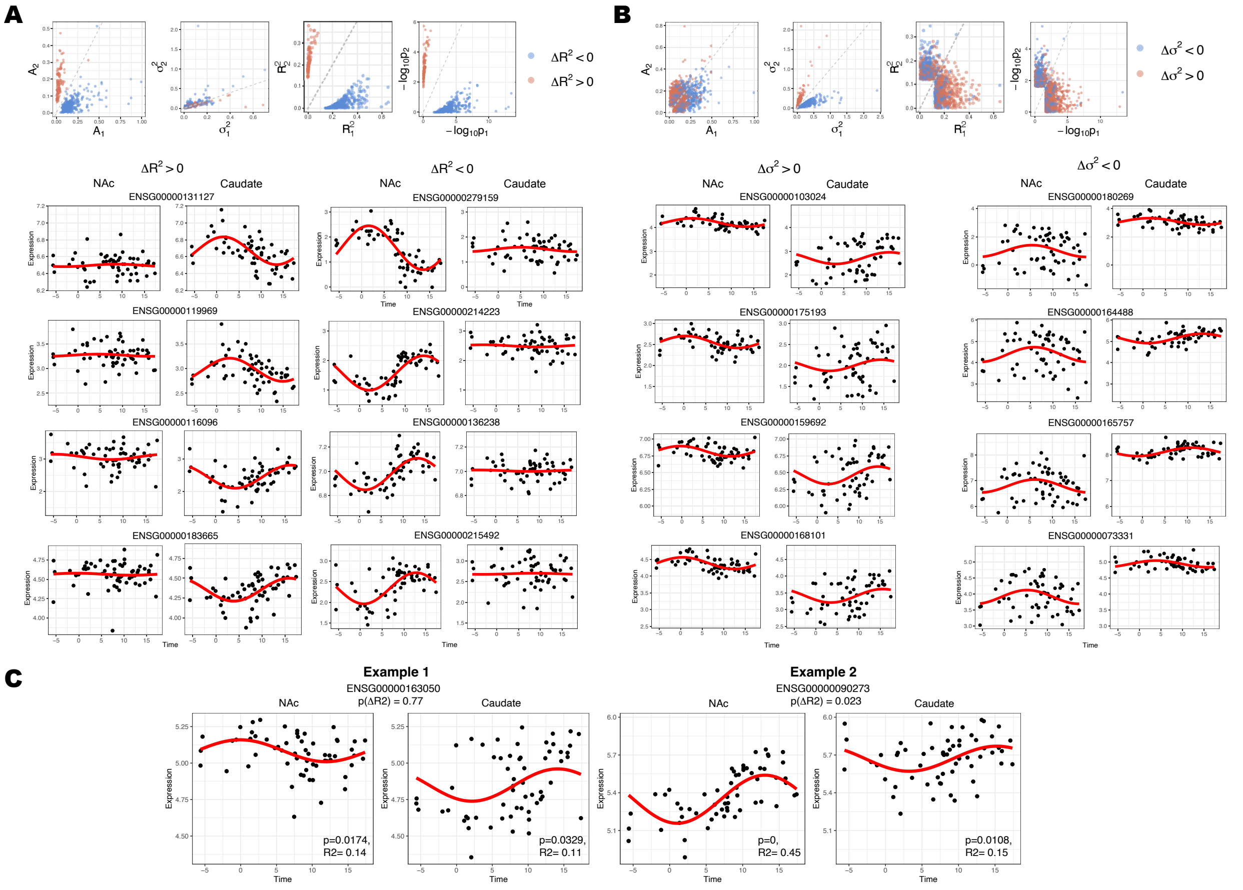

Figure 10: Comparing rhythms between differential R^2 and differential σ^2 genes. A. (Top) Scatter plots of A, σ^2, R^2, and transformed rhythmicity p-values of genes with differential R^2 between NAc and caudate with a cutoff of p-value < 0.05. The gray dashed line is the diagonal reference line. (Bottom) Scatter plots of gene expression (y-axis) and zeitgeber time (x-axis) of the top 4 detected genes in NAc and caudate with $\Delta R^2 > 0$ and $\Delta R^2 < 0$ respectively. B. Same plots as in A using differential σ^2 test. C. Counterexamples for difference between RhyI/RhyII and ΔR^2 genes.

4.1.3 Comparing differential R^2 and differential σ^2 results

Current methods that allow for differential rhythm fitness test for difference in σ^2 (DODR and diffCircadian). However, in real application we found that comparing R^2 is more meaningful than comparing σ^2. Here we justify the decision by comparing the differential R^2 genes and the differential σ^2 genes between NAc and caudate, both with a p-value cutoff of 0.05. As in Figure 10A, an increase in R^2 is almost always associated with larger A and a smaller rhythmicity p-value. Likewise, a decrease in R^2 is almost always associated with smaller A and a larger rhythmicity p-value. Thus we conclude that genes have larger R^2 is more rhythmic. In contrast, a change in σ^2 is not a strong sign of the change of A and rhythmicity p-value. As a result, we test R^2 difference for a strong indicator of change in A and rhythmiciy p-value.

The concept of differential rhythm fitness may look similar to detecting rhythmicity only in one group but not in the other with the SSMS procedure (RhyI/RhyII), but they are for different purposes. We show two counterexamples for the illustration. First, The SSMS procedure is dependent on a dichotomous cutoff, as a result, RhyI/RhyII genes may not have a large difference in rhythm fitness (Figure 10C Example 1). Furthermore, in Figure 10C Example 2, a RhyBoth gene is weakly rhythmic in caudate with a p-value of 0.01 and strongly rhythmic in NAc, which results in a sigficant ΔR^2 (p=0.023).

4.2 Case study two: rhythm comparison between subjects with schizophrenia and unaffected comparison subjects with RNA-Seq data

The CommonMind Consortium (Fromer et al. (2016)) collected RNA-Seq data from dorsolateral prefrontal cortex (dlPFC) of subjects with schizophrenia (SCZ) and unaffected comparison subjects (UC). After preprocessing, we applied the pipeline to 46 pairs of subjects. Each pair consists of one subject with SCZ and one UC subject, matched by sex and age (Seney et al. (2019)).

4.2.1 Identification of types of rhythmicity

With Sidak.FS and a p-value cutoff of 0.05, we identified 268 RhyI genes, 252 RhyII genes, and 12 RhyBoth genes. INGENUITY pathway analysis (IPA) software (Qiagen) was used for pathway enrichment analysis. In UC subjects, the most enriched pathway of the rhythmic genes is the circadian rhythm signaling (p=0.0085), and the top predicted upstream regulators are core clock genes PER1, Arntl-Clock complex, CRY2, PER2, CRY1, NPAS2. While in subjects with SCZ, the most enriched pathways are coronavirus replication pathway (p=0.0023), oxidative phosphorylation (p=0.0083), and cholesterol metabolism pathways. It is intriguing to observe a different set of circadian genes in subjects with SCZ and the common circadian genes with very few RhyBoth genes. As a result, DR parameter test is not applicable in this dataset and only DR fitness test is performed in the next subsection.

4.2.2 Genes with differential rhythm fitness

Next, we performed a differential rhythm fitness test for the 532 genes rhythmic in more than one group. With the likelihood-ratio test and a p-value cutoff of 0.1, we identified 97 genes with a significant change of R^2, where 60 increased R^2 fitness from UC to SCZ and 37 decreased. Calcium signaling is the most enriched pathway for genes with increased R^2 fitness (p=0.0031), which is supported by many studies showing association of elevated activity of Ca^{2+} signaling in subjects with SCZ (Berridge (2013)). The pathway most enriched for genes with decreased R^2 is LPS/IL-1 Mediated Inhibition of RXR Function (p = 0.0027). This observation is corroborated in Vitale et al. (2017), where the same pathway is enriched in the differentially methylated loci between subjects with SCZ and UC subjects in three types of cells (induced pluripotent stem cells, olfactory neurosphere-derived cells, and fibroblasts). Scatter plots of representative genes in LPS/IL-1 (loss of rhythmicity) and calcium signaling (gain of rhythmicity) pathways are shown in Figure 11B.

4.3 Case study three: methylation differential rhythmicity analysis between Snord116$^{+/-}$ and wild type mice

Circadian rhythm exists in many levels of biological activities other than transcriptome. This section applies the pipeline to DNA methylation data to show its generalizability to other omics data. In this study, Snord116-deleted (Snord116$^{+/-}$) mice are contrasted against wild type (WT) mice to study the rhythm change due to the imprinted disorder Prader-Willi syndrome (PWS). For each genotype, cerebral cortex samples are collected at six time points across day, three at each time point ($n_1 = n_2 = 18$). The data is publically available at GEO with the accession number GSE103249.

To increase accuracy and interpretability, We filtered out the methylation measurements at sites where the average sequencing coverage is less than five and aggregated methylation sites into gene regions using the R package methylSig (Park et al. (2014)). Gene annotations were downloaded with UCSC Table Browser (Karolchik et al. (2004)) at http://genome.ucsc.edu. The M values were calculate to fit the cosinor model.

4.3.1 Types of rhythmicity of methylation level by gene

Out of 12,815 methylation regions identified, 271 are RhyI (wild type only) genes, 276 are RhyII (Snord116$^{+/-}$ only) genes, and 15 are RhyBoth under p-value=0.05 cutoff (Figure 12A (left)). We then performed pathway enrichment for genes with rhythmic methylation in WT (RhyI and RhyBoth) and Snord116$^{+/-}$ (RhyII and RhyBoth) separately using Metascape(Figure 12B). The precise role of the genes with rhythmic methylation

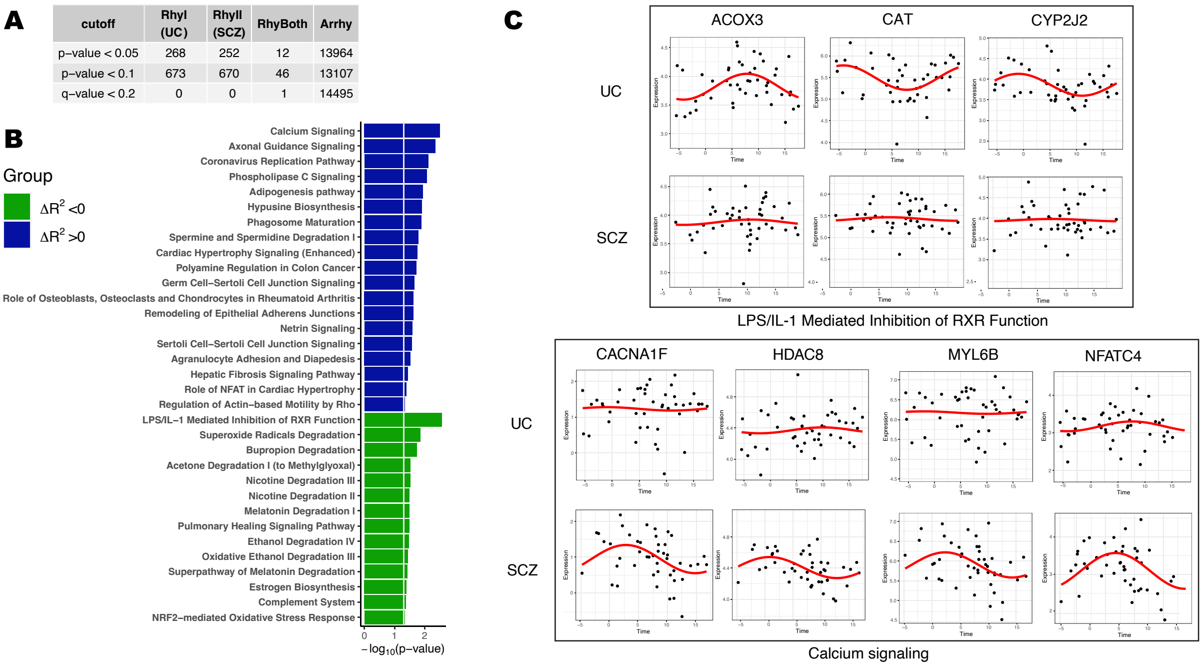

Figure 11: A. Number of genes categorized to each TOR by different cutoffs in Case study two. B. Ingenuity Canonical Pathways enrichement result for genes with R^2 change (p-value<0.1). C. Scatter plots of genes in LPS/IL-1 (loss of rhythmicity) and calcium signaling (gain of rhythmicity) pathways.

patterns in the brain related to PWS is unknown. However, enriched pathways related to female reproduction, including gonad development (p = 0.0036) and oocyte development (p=0.0011) may be associated with hypogonadism, which is commonly found in individuals with PWS.

4.3.2 Differential rhythmicity of methylation level by gene

Since we summarize the methylation level of gene regions, the rhythmicity amplitude does not change much with the transformed M values. As a result, we performed only differential phase test to the 15 RhyBoth genes and found 4 with significant shifted phase ($p < 0.05$): Gm16085, Gm44737, Gm9750, and Pglyrp3.

With DR fitness test, we found 66 genes with significantly changed R^2 in methylation rhythmicity ($p < 0.05$): 33 with increased R^2 fitness in Snord116$^{+/-}$ while 33 have decreased R^2. Figure 12E shows one of the top genes with differential R^2 between the two genotypes (p-value = 0.012), where Utp14b is also associated with male infertility.

4.3.3 Remark on weak signal

The rhythmicity strength of the methylation data is relatively weak.The weak signal could be due to the small sample size ($n = 18$ per group), or the pooling of sequencing read counts of methylation loci in gene regions. However, the TOR categorization procedure only serves as a filtering step to select genes for biologically meaningful DR tests, where the type I error will be further controlled. In fact, we identified 242 significant genes at q-value< 0.5 (Figure 12C). The left-skewed distribution of DRF p-values also demonstrates the DRF signals (Figure 12D).

4.4 Case study four: detecting chronic shift-lag induced rhythm changes in rats using real-time RT-PCR measures

To further demonstrate that the pipeline also applies to low-dimensional data, we applied it to real-time RT-PCR data where mRNA from only five genes was reverse transcribed and quantified. In this study, rats

A

cutoff	RhyI (WT)	RhyII (Snord116+/–)	RhyBoth	Arrhy
p–value < 0.05	271	276	15	12253
p–value < 0.1	511	508	65	11731
q–value < 0.2	0	0	0	12815

B

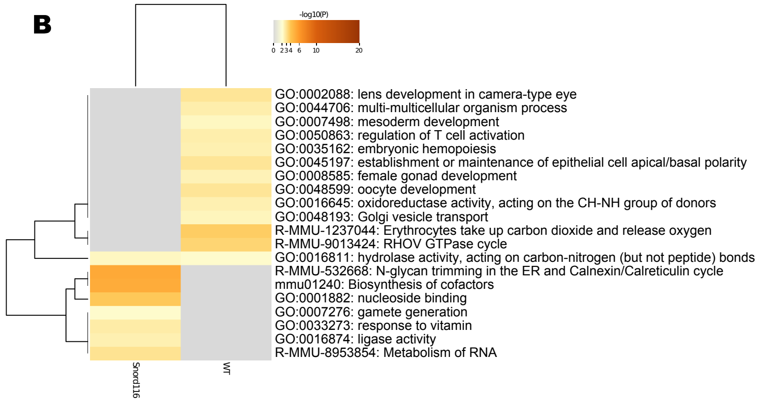

C

test	cutoff	nTRUE
phase	p–value<0.05	4(/15)
DRF	p–value<0.1	187(/562)
DRF	p–value<0.05	66(/562)
DRF	p–value<0.01	7(/562)
DRF	q–value<0.3	242(/562)

D

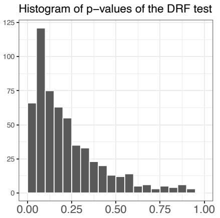

E

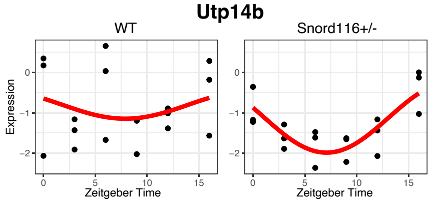

Figure 12: A. Number of genes categorized to each TOR by different cutoffs in Case study three. B. Top pathways enriched for each genotype (p-value<0.05). C. Number of significant genes from differential phase test and differential rhythm fitness (DRF) test by different cutoffs. D. Histogram of DRF test p-vealues. E. Scatter plots of a top ΔR^2 gene Utp14b.

are randomly assigned to two light regimes (n = 36 per lighting regimen): 1) a 12:12 light-dark (LD) cycle, 2) a chronic shift-lag paradigm of 6-hour phase advances occurring every 2 days for 10 repeats. The two groups are then put into DD (completely dark) for 5-7 days until sacrificed at circadian time (CT) 3, 7, 11, 15, 19, and 23 (n=6 per time point). Note that in this study, CT is used instead of ZT, where CT12 is defined as the onset of running-wheel activity. More details of the study design can be found in Logan et al. (2012).

The five quantified genes include two canonical clock genes, Per2 and Bmal1, and three cytokine-related genes IFNγ, perforin and granzyme B. A log2 transformation was performed to the raw measurements to symmetrize the data before the DiffCircaPipeline analyses. A summary of the analyses results and scatter plots for each gene are shown in Figure 13.

4.4.1 Canonical clock genes

The two canonical clock genes are RhyBoth under q<0.05. We further performed the DRF test and DRP (ΔA, $\Delta\phi$, and ΔM) test. Bmal1 has a significantly advanced phase of 8.45 hours (q=3.5e−03) in the shift-lag group. Per2 is significantly changed in all three parameters with an amplitude increase of 0.64 (q=3.5e−05), phase lag of 10.82 hours (q=1.3e−03), and MESOR increase of 0.7 (q=3.2e−08). The rhythm fitness R^2 is also increased by 0.35 by a marginal q-value.

Note that further normalization (e.g. scaling the measurements by maximum value) might eliminate the significant change of A and MESOR in Per2. Nonetheless, the distinguished phase difference suggests that rotating shift workers could suffer a significant disruption in circadian rhythm.

4.4.2 Cytokine-related genes

Among the three cytokine-related genes, only granzyme B is rhythmic in the control group, while IFNγ and perforin are not rhythmic in either condition. As a result, we performed test DRF test on granzyme B and found a decreased R^2 of 0.34 with a marginal q-value of 0.08.

A

Gene	p<0.05	q<0.05	ΔR^2	ΔA	Δϕ	ΔM
Bmal1	RhyBoth	RhyBoth	−0.13(q=0.57)	−0.24(q=0.62)	−8.45(q=3.5e−03)	−0.26(q=0.43)
Per2	RhyBoth	RhyBoth	0.35(q=0.08)	0.64(q=3.5e−05)	10.82(q=1.3e−03)	0.7(q=3.2e−08)
granzyme B	Rhyl	Rhyl	−0.34(q=0.08)	N/A	N/A	N/A
IFN gamma	Arrhy	Arrhy	N/A	N/A	N/A	N/A
perforin	Arrhy	Arrhy	N/A	N/A	N/A	N/A

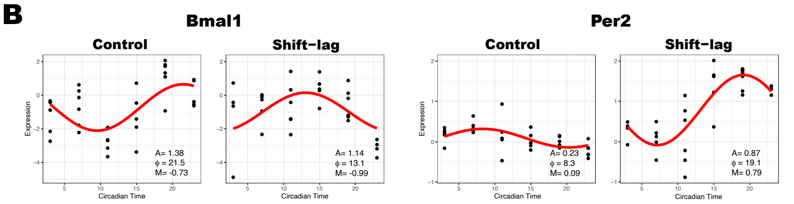

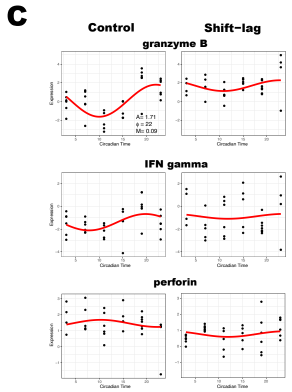

Figure 13: A. a summary table of DiffCircaPipeline results for each gene. B. Scatter plots for two canonical clock genes. C. Scatter plots for three canonical clock genes (only parameters for rhythmic genes are put on the plot).

References

Aho, K., Derryberry, D., and Peterson, T. (2014). Model selection for ecologists: the worldviews of aic and bic. *Ecology*, 95(3):631–636.

Benjamini, Y. and Hochberg, Y. (1995). Controlling the false discovery rate: a practical and powerful approach to multiple testing. *Journal of the Royal statistical society: series B (Methodological)*, 57(1):289–300.

Berridge, M. J. (2013). Dysregulation of neural calcium signaling in alzheimer disease, bipolar disorder and schizophrenia. *Prion*, 7(1):2–13.

Chen, C.-Y., Logan, R. W., Ma, T., Lewis, D. A., Tseng, G. C., Sibille, E., and McClung, C. A. (2016). Effects of aging on circadian patterns of gene expression in the human prefrontal cortex. *Proceedings of the National Academy of Sciences*, 113(1):206–211.

Cornelissen, G. (2014). Cosinor-based rhythmometry. *Theoretical Biology and Medical Modelling*, 11(1):1–24.

Ding, H., Meng, L., Liu, A. C., Gumz, M. L., Bryant, A. J., Mcclung, C. A., Tseng, G. C., Esser, K. A., and Huo, Z. (2021). Likelihood-based tests for detecting circadian rhythmicity and differential circadian patterns in transcriptomic applications. *Briefings in Bioinformatics*, 22(6):bbab224.

Fithian, W., Taylor, J., Tibshirani, R., and Tibshirani, R. (2015). Selective sequential model selection. *arXiv preprint arXiv:1512.02565*.

Fromer, M., Roussos, P., Sieberts, S. K., Johnson, J. S., Kavanagh, D. H., Perumal, T. M., Ruderfer, D. M., Oh, E. C., Topol, A., Shah, H. R., et al. (2016). Gene expression elucidates functional impact of polygenic risk for schizophrenia. *Nature Eeuroscience*, 19(11):1442–1453.

G'Sell, M. G., Wager, S., Chouldechova, A., and Tibshirani, R. (2016). Sequential selection procedures and false discovery rate control. *Journal of the Royal Statistical Society: Series B (Statistical Methodology)*, 78(2):423–444.

Huo, Z., Tang, S., Park, Y., and Tseng, G. (2020). P-value evaluation, variability index and biomarker categorization for adaptively weighted fisher's meta-analysis method in omics applications. *Bioinformatics*, 36(2):524–532.

Karolchik, D., Hinrichs, A. S., Furey, T. S., Roskin, K. M., Sugnet, C. W., Haussler, D., and Kent, W. J. (2004). The ucsc table browser data retrieval tool. *Nucleic Acids Research*, 32(suppl_1):D493–D496.

Ketchesin, K. D., Zong, W., Hildebrand, M. A., Seney, M. L., Cahill, K. M., Scott, M. R., Shankar, V. G., Glausier, J. R., Lewis, D. A., Tseng, G. C., et al. (2021). Diurnal rhythms across the human dorsal and ventral striatum. *Proceedings of the National Academy of Sciences*, 118(2).

Logan, R. W., Zhang, C., Murugan, S., O'Connell, S., Levitt, D., Rosenwasser, A. M., and Sarkar, D. K. (2012). Chronic shift-lag alters the circadian clock of nk cells and promotes lung cancer growth in rats. *The Journal of Immunology*, 188(6):2583–2591.

Marcus, R., Eric, P., and Gabriel, K. R. (1976). On closed testing procedures with special reference to ordered analysis of variance. *Biometrika*, 63(3):655–660.

Park, Y., Figueroa, M. E., Rozek, L. S., and Sartor, M. A. (2014). Methylsig: a whole genome dna methylation analysis pipeline. *Bioinformatics*, 30(17):2414–2422.

Pelikan, A., Herzel, H., Kramer, A., and Ananthasubramaniam, B. (2021). Venn diagram analysis overestimates the extent of circadian rhythm reprogramming. *The FEBS Journal*.

Seney, M. L., Cahill, K., Enwright, J. F., Logan, R. W., Huo, Z., Zong, W., Tseng, G., and McClung, C. A. (2019). Diurnal rhythms in gene expression in the prefrontal cortex in schizophrenia. *Nature Communications*, 10(1):1–11.

Shen, X. and Ye, J. (2002). Adaptive model selection. *Journal of the American Statistical Association*, 97(457):210–221.

Šidák, Z. (1967). Rectangular confidence regions for the means of multivariate normal distributions. *Journal of the American Statistical Association*, 62(318):626–633.

Taylor, J. and Tibshirani, R. J. (2015). Statistical learning and selective inference. *Proceedings of the National Academy of Sciences*, 112(25):7629–7634.

Thaben, P. F. and Westermark, P. O. (2014). Detecting rhythms in time series with rain. *Journal of Biological Rhythms*, 29(6):391–400.

Thaben, P. F. and Westermark, P. O. (2016). Differential rhythmicity: detecting altered rhythmicity in biological data. *Bioinformatics*, 32(18):2800–2808.

Vitale, A. M., Matigian, N. A., Cristino, A. S., Nones, K., Ravishankar, S., Bellette, B., Fan, Y., Wood, S. A., Wolvetang, E., and Mackay-Sim, A. (2017). Dna methylation in schizophrenia in different patient-derived cell types. *npj Schizophrenia*, 3(1):1–11.

Zhou, Y., Zhou, B., Pache, L., Chang, M., Khodabakhshi, A. H., Tanaseichuk, O., Benner, C., and Chanda, S. K. (2019). Metascape provides a biologist-oriented resource for the analysis of systems-level datasets. *Nature Communications*, 10(1):1–10.
